# Responsiveness and Minimal Important Change of the Mini- and Brief-Balance Evaluation Systems Tests in People with Incomplete Cervical Spinal Cord Injury: A Prospective Cohort Study

**DOI:** 10.3390/neurolint17030043

**Published:** 2025-03-18

**Authors:** Yusuke Morooka, Yosuke Kunisawa, Shigeru Obayashi, Yasuyuki Takakura

**Affiliations:** 1 Department of Physical Therapy, Faculty of Health and Medical Care, Saitama Medical University, 981, Kawakado, Iruma-gun 350-0496, Saitama, Japan; kunisawa@saitama-med.ac.jp (Y.K.); takakura@saitama-med.ac.jp (Y.T.); 2 Graduate School of Medicine, Saitama Medical University, 981, Kawakado, Iruma-gun 350-0496, Saitama, Japan; 3 Department of Rehabilitation Medicine, Saitama Medical Center, Saitama Medical University, 1981, Kamoda, Kawagoe 350-8550, Saitama, Japan; ohbayash@saitama-med.ac.jp

**Keywords:** spinal cord injury, minimal important change, postural balance

## Abstract

Background/Objectives: Responsiveness and minimal important change (MIC) are key metrics that vary across conditions and should be determined for specific populations. However, these metrics have not yet been established for the Mini-Balance Evaluation Systems Test (Mini-BESTest) and Brief-BESTest in people with subacute traumatic incomplete cervical spinal cord injury (iCSCI). In this study, we aimed to determine the responsiveness and MIC of the Mini-BESTest and Brief-BESTest in people with subacute iCSCI. Methods: This study included people with iCSCI who could maintain the standing position for 30 s without assistance within 7 days of injury at the university hospital’s advanced critical care center. Responsiveness was assessed by correlating Mini-BESTest and Brief-BESTest change scores with the Berg Balance Scale (BBS). MIC values were determined using the global rating of change scale as an anchor, employing receiver operating characteristic curve methods (MIC_ROC_) and predictive modeling methods adjusted for the proportion of improved participants (MIC_adjusted_). Results: Fifty people with iCSCI were included in the analysis. Changes in BBS scores were moderately positively correlated with changes in Mini-BESTest and Brief-BESTest scores. MIC_adjusted_ values were 3.7 for the Mini-BESTest and 2.2 for the Brief-BESTest. The MIC_ROC_, based on an improvement rate of 64%, was deemed less appropriate for interpreting meaningful changes due to the high proportion of improved participants. Conclusions: MIC_adjusted_ benchmarks can help clinicians measure significant improvements in dynamic balance, design effective interventions, and evaluate rehabilitation outcomes in people with iCSCI.

## 1. Introduction

The number of people with traumatic incomplete cervical spinal cord injury (iCSCI) classified as grade D based on the American Spinal Injury Association Impairment Scale (AIS) is increasing [1]. This trend is due to increased injuries, such as falls from any height and on level surfaces, among middle-aged and older people owing to the aging population [1]. Physical therapy (PT) for subacute iCSCI, classified as AIS grade D, focuses on regaining walking ability [2]. Walking ability is closely related to balance [3], highlighting the importance of dynamic balance ability [4,5,6,7]. Reactive stepping responses [4], changes in sensory input [5,7], and dual-task conditions [6] may further impair balance, suggesting a multifaceted impact of spinal cord injury (SCI) on balance [8].

The Berg Balance Scale (BBS) is the most widely used balance assessment tool for people with incomplete SCI, with excellent clinical utility [8]; however, it does not assess reactive postural control or gait balance and is prone to ceiling effects [9]. Therefore, a shortened version of the Balance Evaluation Systems Test (BESTest), such as the Mini-BESTest, is considered the most comprehensive dynamic balance assessment tool [8]. The shortened versions include the Mini-BESTest [10] and Brief-BESTest [11]. Their responsiveness [12,13,14,15] and minimal important change (MIC) [12,13,14,15,16,17] have been reported in various disorders.

Responsiveness is the ability of a measurement instrument to detect changes in the construct to be measured [18]. In acute to subacute iCSCI, the degree of recovery can be significant [19]; therefore, specific reference values, such as the MIC, should be utilized for effective clinical interpretation [20]. MIC represents the minimum threshold of difference or changes judged as meaningful or valuable [21,22]. This reference is crucial for demonstrating clinical rather than statistical significance when determining treatment effectiveness [20,23]. Using MIC for clinical interpretation enhances decision making and the development of effective interventions. Responsiveness and MIC are condition-specific and should be evaluated in specific populations [24]. To the best of our knowledge, no studies have reported the responsiveness and MIC of the Mini-BESTest and Brief-BESTest in people with subacute traumatic iCSCI [25,26]. This study aimed to establish the responsiveness and MIC of the Mini-BESTest and Brief-BESTest as dynamic balance assessment tools in people with subacute traumatic iCSCI.

## 2. Materials and Methods

### 2.1. Study Design, Participants, and Ethics

In this single-center prospective cohort study, the following enrollment criteria were applied: (1) people with traumatic iCSCI admitted to the Advanced Critical Care Center of the hospital from November 2022 to July 2024; (2) no psychiatric disorders or dementia that would affect their understanding of the examination procedures and instructions; (3) age between 20 and 90 years; and (4) ability to maintain the standing position without hand support or assistance for more than 30 s within 7 days of injury. The exclusion criteria were as follows: (1) motor dysfunction due to cerebrovascular disease or incurable neurological disease; (2) trauma or fracture of the pelvis or lower extremities; (3) hospitalization for less than 10 days; and (4) pain due to osteoarthritis of the hip or knee during activities of daily living (ADL). These criteria were assessed based on a review of medical records. The presence and type of pain before iCSCI were evaluated during medical interviews.

This study was conducted according to the Strengthening the Reporting of Observational Studies in Epidemiology statement [27]. This study was approved by the Research Ethics Committee of Saitama Medical Center (Approval No. SOU 2022-079) and was conducted according to the Declaration of Helsinki. All participants were informed orally and in writing about the nature of the study and provided written consent to participate before study initiation.

### 2.2. Outcome Measures

A baseline assessment was performed within 7 days post-injury, and a follow-up assessment was performed at discharge. Balance assessments were typically performed on the same day; however, if necessary, the remaining assessments were conducted the following day based on the participant’s condition [14]. Different independent assessors conducted the BBS and Mini-BESTest or the Brief-BESTest.

The Mini-BESTest consists of 14 items and four elemental functions: anticipatory postural control, reactive postural response, sensory orientation, and stability in gait. Each item is scored on a 3-point scale (0: severe to 2: normal), with a total score of 28 points [10]. The Brief-BESTest consists of six elemental functions: biomechanical constraints, verticality and stability limits, anticipatory postural control, reactive postural response, sensory orientation, and stability in gait. Each item is scored on a 4-point scale (0: severe to 3: normal). Of the six items, two were scored separately for the left and right sides, totaling eight items and 24 points [11]. Three physical therapists certified in SCI (≥10 years of experience) who were not directly involved in their PT sessions conducted the Mini-BESTest and Brief-BESTest scores in a single trial. The evaluations were distributed among evaluators based on their availability. The reliability of these test evaluations by the raters was satisfactory [28]. Walking aids, if needed, were permitted during the assessment of gait parameters. If walking assistance was required, the item was rated as impossible (0: severe).

The BBS consists of 14 movement tasks, including sitting and standing, rated on a 5-point scale (from 0: unable to perform or need help to 4: normal performance), with a total score of 56 points [29]. The BBS is highly responsive in people with incomplete SCI [30,31]. The assessments were scored by an assigned physical therapist in a single trial.

The Global Rating of Change (GRC) scale assessed whether the participants’ conditions improved or worsened. The GRC is a 7-point scale with ratings of much better (+3), moderately better (+2), slightly better (+1), no change (0), slightly worse (–1), moderately worse (–2), and much worse (–3) [32]. The GRC has demonstrated adequate validity and responsiveness [32,33]. The evaluator was a physical therapist who was not directly involved in their PT sessions and provided the participants with written and verbal explanations of the GRC scale. At discharge, the participants were asked, “How has your balance changed since the initial assessment?” Based on their judgment, participants were assigned to the “improved” group if their GRC score was ≥2 and to the “unimproved” group if it was <2. Moderate or more significant improvements were considered meaningful [33]. As reported previously [19], people with iCSCI classified as AIS grade D in the subacute phase have a significant potential for recovery. Considering this, a moderately better (+2) or higher value can be interpreted as a meaningful improvement in this population.

### 2.3. Data Collection

The participants’ medical records included information on age, sex, AIS grade, neurological level of injury, causes of injury, number of days from injury to rehabilitation, number of days from injury to assessment, number of days between baseline and follow-up (at discharge) assessments, upper and lower extremity motor scores, and type of walking aid used.

### 2.4. Rehabilitation

All participants underwent 40 min of PT five times a week during hospitalization. Each participant with iCSCI aimed to reestablish their gait, including early release from bed, basic movement exercises, standing balance exercises, and gait exercises. Occupational therapy was provided for upper extremity function and ADL when necessary. The rehabilitation content was not controlled because the primary purpose of this study was to investigate important changes in balance ability.

### 2.5. Data Analysis

Statistical analysis was performed using R version 4.0.2 (R Foundation for Statistical Computing, Vienna, Austria), with a significance level of 5%. Descriptive statistics were computed, including participant and dropout characteristics and balance scores. Continuous variables are presented as means with standard deviations, and categorical variables are presented as counts with percentages. Before conducting statistical analyses, normality was assessed using the Shapiro–Wilk test. As normality was confirmed only for the Mini-BESTest score comparison between the improved and unimproved groups, a parametric (unpaired *t*-test) was used. Non-parametric methods were applied for all other analyses: the Mann–Whitney U test was used for Brief-BESTest group comparisons, and Wilcoxon signed-rank tests were used for within-group comparisons. Spearman’s correlation analysis was used for all correlation tests. Responsiveness was evaluated by calculating Spearman’s rank correlation of changes in Mini-BESTest and Brief-BESTest scores with changes in BBS scores. Correlation coefficients of less than 0.10 were interpreted as negligible, 0.10–0.39 as weak, 0.40–0.69 as moderate, 0.70–0.89 as strong, and 0.90–1.0 as very strong [34]. The responsiveness hypothesis was based on previous studies [15] on the Mini-BESTest. Although we found no studies that directly compared changes in the Brief-BESTest scores with those in the BBS scores, we assumed that the Mini-BESTest and Brief-BESTest score changes were positively correlated with the BBS score changes to a greater than a moderate degree (ρ ≥ 0.4).

The MIC values were determined using GRC as an anchor, employing receiver operating characteristic curve methods (MIC_ROC_) [35] and predictive modeling methods (MIC_predict_) [21,36]. As a preliminary check, Spearman’s rank correlation coefficient was calculated for the correlation between GRC and changes in the Mini-BESTest and Brief-BESTest scores. To estimate MICs, a correlation coefficient of ≥0.3 between the anchor and the change score on the instrument of interest should be selected [37]. Therefore, correlation coefficients of ≥0.3 were used in this study. For the MIC_ROC_, ROC curves were used to calculate the optimal cutoff value for distinguishing improved and unimproved participants by GRC based on the amount of change in Mini-BESTest and Brief-BESTest. To evaluate the discriminative ability of this cutoff, the sensitivity, specificity, area under the curve (AUC), and 95% confidence interval (CI) of the AUC were calculated, with the AUC used to assess discrimination accuracy. An AUC ≥0.7 implied sufficient discrimination accuracy [38]. MIC_predict_ was determined by logistic regression analysis with Mini-BEST and Brief-BEST change scores as independent variables and dichotomized GRCs (improved/unimproved) as the dependent variable [21]. MIC_predict_ is defined as the change score for which the likelihood ratio equals 1 [21]. This method is considered more accurate than the MIC_ROC_ approach because it is less affected by random sampling. Bias can be adjusted if the proportion of participants who improved is not 50%. The predictive modeling methods adjusted for the proportion of improved participants (MIC_adjusted_) can be calculated if the proportions of improved and unimproved participants differ, allowing for a more accurate estimate of the MIC value [36]. Therefore, the MIC_adjusted_ value was considered the most statistically accurate in this study. A visual anchor-based MIC distribution was created by combining and integrating anchor- and distribution-based approaches [39]. This provides a visual representation of the distribution curve in terms of the percentage change in the outcome for participants who achieved a significant improvement in the anchor (GRC ≥ 2) and those who did not (GRC < 2) [37,39].

According to the COSMIN checklist [40] and expert-proposed quality criteria [38], a sample size of at least 50 participants is recommended to validate responsiveness and MIC. Therefore, the target number of participants in this study was 50.

## 3. Results

### 3.1. Participant Characteristics

**Figure 1:**
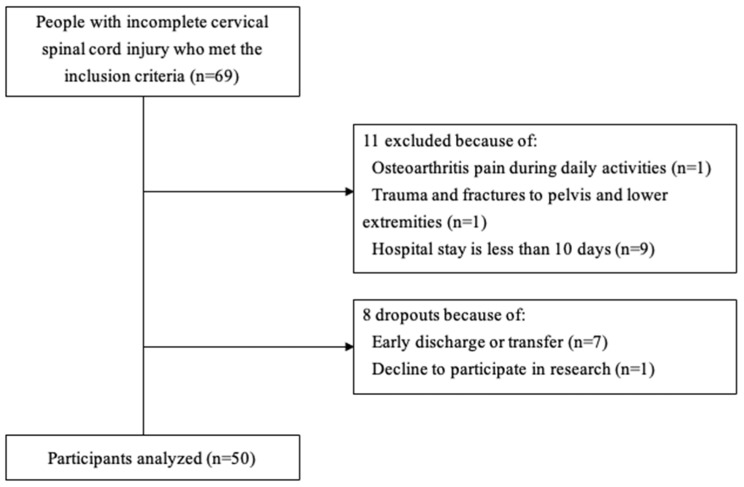
Flow diagram of participant recruitment and inclusion in the study.

The participants’ characteristics are shown in Table 1. A total of 69 participants were enrolled, of whom 11 did not meet the inclusion/exclusion criteria and 8 dropped out due to withdrawal from the study, emergency discharge, or transfer to another hospital, leaving a total of 50 participants (Figure 1). The number of days between baseline and follow-up was 15.3 ± 8.7 days. Data on balance at baseline and follow-up are presented in Table 2. Based on GRCs in each test, 32 (64%) showed improvement, while 18 (36%) did not show improvement. The Mini-BESTest and Brief-BESTest changes were significantly greater in the improved group than in the unimproved group (*p* < 0.05).

### 3.2. Responsiveness

**Figure 2:**
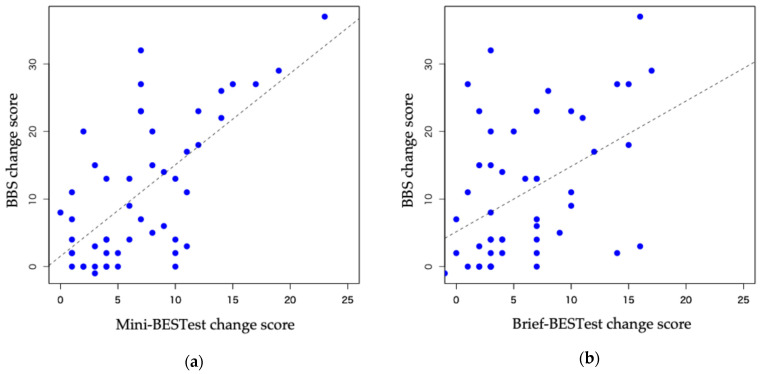
Correlations between the BBS change scores with Mini-BESTest and Brief-BESTest change scores. (**a**) Mini-BESTest (ρ = 0.57, 95% CI: 0.47–0.80); (**b**) Brief-BESTest (ρ = 0.42, 95% CI: 0.19–0.65). BBS: Berg Balance Scale; BESTest: Balance Evaluation System Test; CI: confidence interval.

The correlations of BBS scores and Mini-BESTest and Brief-BESTest scores were moderately significant (ρ = 0.57, *p* < 0.05 and ρ = 0.42, *p* < 0.05, respectively) (Figure 2).

### 3.3. MIC

**Figure 3:**
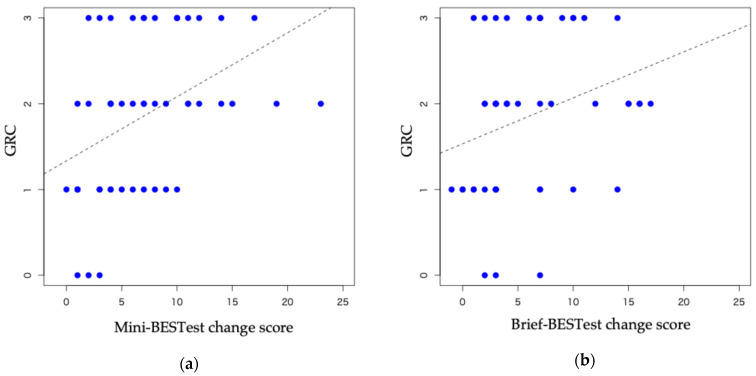
Correlations between the GRC and Mini-BESTest and Brief-BESTest change scores. Correlations between GRC and (**a**) Mini-BESTest (ρ = 0.48, 95% CI: 0.16–0.63); (**b**) Brief-BESTest (ρ = 0.35, 95% CI: 0.01–0.52). BESTest: Balance Evaluation Systems Test; CI: confidence interval; GRC: Global Rating of Change scale.

The correlation between GRC and changes in test scores was moderately significant for the Mini-BESTest (ρ = 0.48, *p* < 0.05) and weak but significant for the Brief-BESTest (ρ = 0.35, *p* < 0.05) (Figure 3). GRC was the anchor in both tests because the correlation coefficient was >0.3.

**Figure 4:**
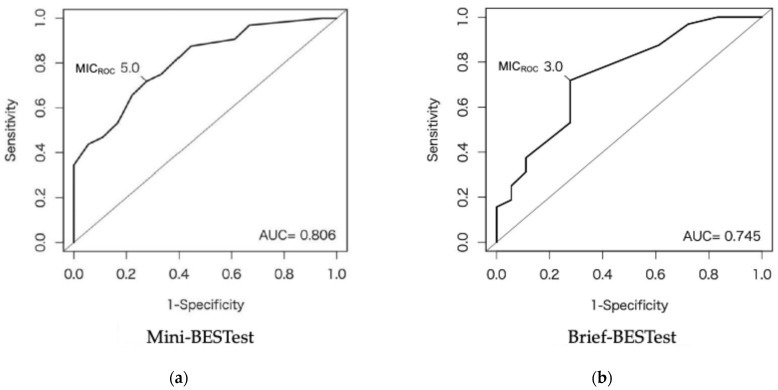
ROC curves for the change in the Mini-BESTest and Brief-BESTest scores based on the GRC. (**a**) ROC curve for the Mini-BESTest (MIC_ROC_ = 5.0, AUC: 0.81, 95% CI: 0.69–0.93); (**b**) ROC curve for the Brief-BESTest (MIC_ROC_ = 3.0, AUC: 0.75, 95% CI: 0.60–0.89). AUC: Area under the curve; BESTest: Balance Evaluation System Test; CI: confidence interval; MIC: minimal important change; ROC: receiver operating characteristic.

**Figure 5:**
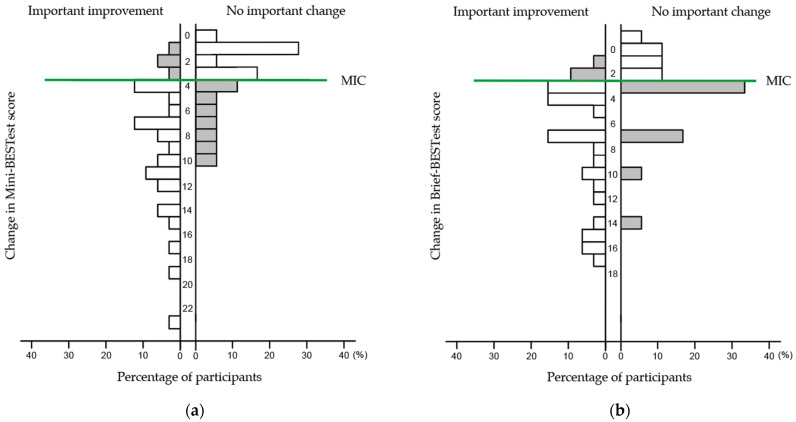
Distribution of Mini-BESTest and Brief-BESTest change scores (percentages) for the improved and unimproved groups: (**a**) Mini-BESTest; (**b**) Brief-BESTest. The left and right sides of the figure show the distribution of change in each balance assessment for improved and unimproved balances, respectively. The MIC values in the figure indicate MIC_adjusted_, rounded to the nearest whole number for clinical relevance (Mini-BESTest: 4, Brief-BESTest: 3). BESTest: Balance Evaluation System Test; MIC: minimal important change.

The MIC_ROC_ with GRC as the anchor were 5.0 (AUC: 0.81, 95% CI: 0.69–0.93) for the Mini-BESTest and 3.0 (AUC: 0.75, 95% CI: 0.60–0.89) for the Brief-BESTest (Table 3, Figure 4). The MIC_predict_ values for Mini-BESTest and Brief-BESTest were 4.1 and 2.6, respectively. These values were adjusted because the proportion of participants who “improved” was 64%, not 50%. After adjustment, the MIC_adjusted_ values were 3.7 and 2.2 for Mini-BESTest and Brief-BESTest, respectively. The anchor-based MICs exceeded 36 participants (72%) for the Mini-BESTest and 39 participants (78%) for the Brief-BESTest. The visual anchor-based MIC for the Mini-BESTest and Brief-BESTest are shown in Figure 5. The left and right sides of the figure show the distribution of change in each balance assessment for improved and unimproved balance, respectively. The distribution of the change in balance assessment between the improved and unimproved participants differed.

## 4. Discussion

This is the first study to evaluate the responsiveness and MIC of the Mini-BESTest and Brief-BESTest in people with subacute iCSCI. The ability to adequately measure changes in individual performance could be an effective assessment tool for clinical practice. Changes in balance ability were assessed using the Mini-BESTest and Brief-BESTest and were moderately positively correlated with those assessed using the BBS. This finding supports our hypothesis; as a previous study [15] found that in people with balance disorders, there was a moderately positive correlation (*r* = 0.58) between changes in the Mini-BESTest and BBS scores.

In our study, the MIC_ROC_, MIC_predict_, and MIC_adjusted_ were 5.0, 4.1, and 3.7 in the Mini-BESTest and 3.0, 2.6, and 2.2 in the Brief-BESTest, respectively. Both MIC_ROC_ values demonstrated sufficient discriminatory ability [38], with a sensitivity of 71.9%, a specificity of 72.2%, and an AUC exceeding 0.7. ROC-based and predictive modeling methods are known to bias and overestimate MIC when the improvement rates based on anchors exceed 50% [36]. When the proportion of improved participants deviates from 50%, MIC_predict_ can be adjusted to MIC_adjusted_, which provides a more accurate estimate by accounting for the imbalance in group proportions [36]. In this study, MIC_adjusted_ was lower than MIC_predict_, supporting previous findings that MIC_predict_ tends to overestimate MIC, particularly when the proportion of improved participants exceeds 50%. Therefore, MIC_adjusted_ was considered the most appropriate parameter for this study. In this study, where the improvement rate was 64%, the MIC_predict_ values were adjusted, and the MICs of Mini-BESTest and Brief-BESTest were interpreted as 3.7 and 2.2, respectively. Previous studies have reported an MIC in the Mini-BESTest ranging from 1.5 to 4.0 for various diseases, including stroke, Parkinson’s disease, balance disorders, and total knee arthroplasty [13,14,15,16,17]. In this study, the MIC of Mini-BESTest was 3.7, which falls within this range. However, the MIC values in previous studies were calculated using ROC-based methods. The MIC_ROC_ in this study was 5.0, which exceeded this range. In addition to being influenced by sample bias, this result is likely due to the timing of assessment (acute to subacute phase) and the characteristics of the disease (high possibility of recovery). In the acute-to-subacute phase for people with iCSCI, rapid functional recovery can lead to greater score changes [19] and contribute to the higher MIC_ROC_ observed in this study. In contrast, we used predictive modeling to calculate MIC_adjusted_, which accounts for sample bias and was considered a more accurate estimate. This suggests that MIC_adjusted_ is a more reliable and appropriate parameter for evaluating the effects of balance improvement. A study found that the MIC of the Brief-BESTest in people with subacute stroke was 4.5 points [14]. In contrast, the MIC of this test in other conditions was reported as 3.0–3.3 points [12,17]. Given that the latter studies used different anchors, the MIC obtained in the former study should be considered a more relevant reference. This difference could be attributed to cohort characteristics, particularly the presence of bilateral paralysis in iCSCI. The Brief-BESTest assesses anticipatory and reactive postural control on both sides of the body, which may be more strongly influenced by bilateral paralysis. In the present study, the value was even lower at 2.2, which may be explained by the fact that it is an MIC_adjusted_ value. Given that anchor-based MIC can vary by specific population, calculating the MIC for the Mini-BESTest and Brief-BESTest in people with subacute iCSCI is crucial. We calculated the MIC based on the judgment of participants with iCSCI classified as AIS grade D, who, despite standing for at least 30 s, face challenges in sustained balance and walking tasks. This participant-based perspective is crucial for evaluating MIC [22,41]. In contrast, recall bias is a potential concern when using the GRC scale as an anchor to determine MIC. However, since the typical length of stay in acute care hospitals in Japan is approximately 2-weeks [42], this timeframe seems sufficient for evaluating MIC, with minimal concerns about recall bias [32].

In the visual anchor-based MIC distribution (Figure 5), we confirmed that the distributions of the Mini-BESTest and Brief-BESTest scores for improved and unimproved participants differed. The MIC for the anchor-based method considers the statistical characteristics derived from the distribution [36]; however, it is challenging to examine the distribution itself based on its features [39]. We evaluated the usefulness of MIC by visually assessing the peaks of change by assessing the distribution. In the Mini-BESTest, approximately 60% of the unimproved participants showed a change of 0–3 points, with a peak at 1 point. Conversely, approximately 90% of the improved participants showed a change of 4 points above. This suggests that the Mini-BESTest MIC is appropriate. The Brief-BESTest had a peak of 3 points for the improved and unimproved groups, with an exceptionally high peak in the unimproved group, where approximately 30% of participants were distributed. A change of 3 points in the Brief-BESTest does not always indicate improvement and should be interpreted cautiously. However, approximately 40% of the unimproved participants showed a change of 0–2 points, and approximately 90% of the improved participants showed a change of ≥3 points, which is useful. Based on these distributions, the MIC values of these tests suggest their applicability for people with subacute iCSCI. The Mini-BESTest demonstrated greater discriminatory ability in this population, aligning with previous findings in people who underwent knee arthroplasty [17]. In contrast, while the Brief-BESTest exhibited a less pronounced separation between improved and unimproved participants, its shorter administration time makes it a feasible option for clinical settings where efficiency is a priority.

This study had some limitations. First, participants were recruited using a serial sampling method from a single center, which might have reduced external validity. Second, MIC may vary according to the participant and treatment characteristics [24]. Our study population consisted of people with subacute iCSCI classified as AIS grade D who did not require hand support or assistance for at least 30 s, which limits the generalizability of MIC to people with other injury severities and neurological levels. Finally, because the GRC assesses subjective degrees of improvement, it may represent judgments about changes other than those measured by the study’s variables [33]. Therefore, further research incorporating multiple anchors, including the GRC and objective information, is needed to determine a more appropriate MIC.

## 5. Conclusions

MIC_adjusted_ values are useful for assessing improvements in dynamic balance in people with subacute iCSCI. When the Mini-BESTest and Brief-BESTest scores show a change of ≥4.0 and 3.0, respectively, this can be interpreted as the minimum meaningful change in the individual’s balance ability. These benchmarks will help clinicians detect meaningful improvements in balance in people with subacute iCSCI, guiding intervention effectiveness and rehabilitation strategies in clinical practice.

## Figures and Tables

**Table 1:** Participant characteristics.

	Participants (*n* = 50)	Dropouts (*n* = 19)
Sex (Males/Females)	37/13	15/4
Age (years)	68.3 (13.4)	69.6 (9.3)
AIS (A,B,C/D)	0/50	0/19
NLI		
C4	9 (18.0)	3 (15.8)
C5	13 (26.0)	4 (21.1)
C6	5 (10.0)	3 (15.8)
C7	5 (10.0)	1 (5.3)
C8	18 (36.0)	8 (42.1)
Causes of injury		
Falls on level surface	20 (40.0)	6 (31.6)
Fall from a height	16 (32.0)	4 (21.1)
Traffic accident	5 (10.0)	2 (10.5)
Other	9 (18.0)	7 (36.4)
Time from injury to rehabilitation (days)	3.2 (1.4)	2.8 (1.3)
Time from injury to assessment (days)	5.0 (1.5)	4.6 (1.5)
Between assessment (days)	15.3 (8.7)	–
UEMS (0–50)	41.0 (9.7)	43.8 (6.6)
LEMS (0–50)	48.1 (3.0)	48.3 (3.0)
Walking aid (Walker/None)	23/27	4/15
BBS (0–56)	40.3 (15.1)	46.3 (10.2)

Data are presented as means (standard deviations) or numbers (percentages), depending on the variable type. AIS: American Spinal Injury Association Impairment Scale; BBS: Berg Balance Scale; LEMS: lower extremity motor score; NLI: neurological level of injury; UEMS: upper extremity motor score.

**Table 2:** Baseline and follow-up assessment of each balance.

	Group	Baseline	Follow-Up	Change Score	*p*-Value	
					Baseline vs. Follow-Up	Improved vs. Unimproved
Mini-BESTest	Improved (*n* = 32)	13.5 (7.2)	22.3 (5.5)	8.8 (5.2)	<0.05 *	<0.05 ^†^
	Unimproved (*n* = 18)	15.5 (7.6)	19.3 (7.3)	3.8 (3.1)	<0.05 *	
Brief-BESTest	Improved (*n* = 32)	8.2 (7.0)	15.6 (6.1)	7.4 (4.9)	<0.05 *	<0.05 ^‡^
	Unimproved (*n* = 18)	8.6 (6.9)	12.4 (8.2)	3.8 (3.8)	<0.05 *	

All values are presented as the mean (standard deviation). *: Wilcoxon signed-rank tests; ^†^: Unpaired *t*-test; ^‡^: Mann–Whitney U test; BESTest: Balance Evaluation System Test.

**Table 3:** MIC values in the Mini-BESTest and Brief-BESTest.

	Mini-BESTest	Brief-BESTest
MIC_ROC_	5.0	3.0
AUC (95% CI)	0.81 (0.69–0.93)	0.75 (0.60–0.89)
Sensitivity	71.9	71.9
Specificity	72.2	72.2
MIC_predict_	4.1	2.6
MIC_adjusted_	3.7	2.2

AUC: area under the curve; BESTest: Balance Evaluation System Test; CI: confidence interval; MIC: minimal important change.

## Data Availability

The datasets generated and analyzed on the page are not publicly available but are available from the corresponding author on reasonable request.

## References

[1] Miyakoshi N., Suda K., Kudo D., Sakai H., Nakagawa Y., Mikami Y., Suzuki S., Tokioka T., Tokuhiro A., Takei H. (2021). A Nationwide Survey on the Incidence and Characteristics of Traumatic Spinal Cord Injury in Japan in 2018. Spinal Cord.

[2] Scivoletto G., Tamburella F., Laurenza L., Torre M., Molinari M. (2014). Who is Going to Walk? A Review of the Factors Influencing Walking Recovery after Spinal Cord Injury. Front. Hum. Neurosci..

[3] Lemay J.F., Noamani A., Unger J., Houston D.J., Rouhani H., Musselmann K.E. (2021). Using Wearable Sensors to Characterize Gait After Spinal Cord Injury: Evaluation of Test-Retest Reliability and Construct Validity. Spinal Cord.

[4] Lee J.W., Mauceri S., Chan K., Unger J., Musselman K.E., Masani K. (2023). Stepping Responses for Reactive Balance for Individuals with Incomplete Spinal Cord Injury. J. Biomech..

[5] Noamani A., Lemay J.F., Musselman K.E., Rouhani H. (2021). Characterization of Standing balance after incomplete Spinal Cord Injury: Alteration in Integration of Sensory Information in Ambulatory Individuals. Gait Posture.

[6] Tse C.M., Carpenter M.G., Liu-Ambrose T., Chisholm A.E., Lam T. (2017). Attentional Requirements of Postural Control in People with Spinal Cord Injury: The Effect of Dual Task. Spinal Cord.

[7] Lemay J.F., Gagnon D., Duclos C., Grangeon M., Gauthier C., Nadeau S. (2013). Influence of Visual Inputs on Quasi-Static Standing Postural Steadiness in Individuals With Spinal Cord Injury. Gait Posture.

[8] Arora T., Oates A., Lynd K., Musselman K.E. (2020). Current State of Balance Assessment during Transferring, Sitting, Standing and Walking Activities for the Spinal Cord Injured Population: A Systematic Review. J. Spinal Cord Med..

[9] Jørgensen V., Opheim A., Halvarsson A., Franzén E., Roaldsen K.S. (2017). Comparison of the Berg Balance Scale and the Mini-BESTest for Assessing Balance in Ambulatory People with Spinal Cord Injury: Validation Study. Phys. Ther..

[10] Franchignoni F., Horak F., Godi M., Nardone A., Giordano A. (2010). Using Psychometric Techniques to Improve the Balance Evaluation Systems Test: The Mini-BESTest. J. Rehabil. Med..

[11] Padgett P.K., Jacobs J.V., Kasser S.L. (2012). Is the BESTest at its Best? A Suggested Brief Version Based on Interrater Reliability, Validity, Internal Consistency, and Theoretical Construct. Phys. Ther..

[12] Paixão C., Rebelo P., Oliveira A., Jácome C., Cruz J., Martins V., Simão P., Marques A. (2021). Responsiveness and Minimal Clinically Important Difference of the Brief-BESTest in People With COPD After Pulmonary Rehabilitation. Phys. Ther..

[13] Godi M., Arcolin I., Giardini M., Corna S., Schieppati M. (2020). Responsiveness and Minimal Clinically Important Difference of the Mini-BESTest in Patients with Parkinson’s Disease. Gait Posture.

[14] Winairuk T., Pang M.Y.C., Saengsirisuwan V., Horak F.B., Boonsinsukh R. (2019). Comparison of Measurement Properties of Three Shortened Versions of the Balance Evaluation System Test (BESTest) in People with Subacute Stroke. J. Rehabil. Med..

[15] Godi M., Franchignoni F., Caligari M., Giordano A., Turcato A.M., Nardone A. (2013). Comparison of Reliability, Validity, and Responsiveness of the Mini-BESTest and Berg Balance Scale in Patients with Balance Disorders. Phys. Ther..

[16] Beauchamp M.K., Niebuhr R., Roche P., Kirkwood R., Sibley K.M. (2021). A Prospective Study to Establish the Minimal Clinically Important Difference of the Mini-BESTest in Individuals with Stroke. Clin. Rehabil..

[17] Chan A.C., Pang M.Y., Ouyang H., Jehu D.A. (2020). Minimal Clinically Important Difference of Four Commonly Used Balance Assessment Tools in Individuals after Total Knee Arthroplasty: A Prospective Cohort Study. PM R.

[18] Mokkink L., Terwee C., de Vet H. (2021). Key Concepts in Clinical Epidemiology: Responsiveness, the Longitudinal Aspect of Validity. J. Clin. Epidemiol..

[19] Fawcett J.W., Curt A., Steeves J.D., Coleman W.P., Tuszynski M.H., Lammertse D., Bartlett P.F., Blight A.R., Dietz V., Ditunno J. (2007). Guidelines for the Conduct of Clinical Trials for Spinal Cord Injury as Developed by the ICCP Panel: Spontaneous Recovery after Spinal Cord Injury and Statistical Power Needed for Therapeutic Clinical trials. Spinal Cord.

[20] Angst F., Aeschlimann A., Angst J. (2017). The Minimal Clinically Important Difference Raised the Significance of Outcome Effects Above the Statistical Level, with Methodological Implications for Future Studies. J. Clin. Epidemiol..

[21] Terluin B., Eekhout I., Terwee C.B., de Vet H.C. (2015). Minimal Important Change (MIC) Based on a Predictive Modeling Approach was More Precise than MIC Based on ROC Analysis. J. Clin. Epidemiol..

[22] Jaeschke R., Singer J., Guyatt G.H. (1989). Measurement of Health Status. Ascertaining the Minimal Clinically important Difference. Control Clin. Trials.

[23] Elkins M.R., Pinto R.Z., Verhagen A., Grygorowicz M., Söderlund A., Guemann M., Gómez-Conesa A., Blanton S., Brismée J.M., Agarwal S. (2022). Statistical Inference Through Estimation: Recommendations from the International Society of Physiotherapy Journal Editors. Braz. J. Phys. Ther..

[24] Revicki D., Hays R.D., Cella D., Sloan J. (2008). Recommended Methods for Determining Responsiveness and Minimally Important Differences for Patient-Reported Outcomes. J. Clin. Epidemiol..

[25] Meseguer A.B., Lopez J.A., Lopez J.J., Martinez I. (2025). Psychometric properties of the Mini-Balance Evaluation Systems Test (Mini-BESTest) among multiple populations: A COSMIN systematic review and meta-analysis. Disabil. Rehabil..

[26] Lo C.W.T., Lin C.Y., Tsang W.W.N., Yan C.H., Wong A.Y.L. (2022). Psychometric Properties of Brief-Balance Evaluation Systems Test Among Multiple Populations: A Systematic Review and Meta-analysis. Arch. Phys. Med. Rehabil..

[27] von Elm E., Altman D.G., Egger M., Pocock S.J., Gøtzsche P.C., Vandenbroucke J.P. (2008). The Strengthening the Reporting of Observational Studies in Epidemiology (STROBE) Statement: Guidelines for Reporting Observational Studies. J. Clin. Epidemiol..

[28] Morooka Y., Takakura Y., Kunisawa Y., Okubo Y., Araki S., Obayashi S. (2024). Reliability of the Mini-BESTest and Brief-BESTest for Assessing Patients with Incomplete Spinal Cord Injury. Spinal Cord.

[29] Berg K. (1989). Measuring Balance in the Elderly: Preliminary Development of an Instrument. Physiother. Can..

[30] Tamburella F., Scivoletto G., Iosa M., Molinari M. (2014). Reliability, Validity, and Effectiveness of Center of Pressure Parameters in Assessing Stabilometric Platform in Subjects with Incomplete Spinal Cord Injury: A Serial Cross-sectional Study. J. Neuroeng. Rehabil..

[31] Datta S., Lorenz D.J., Harkema S.J. (2012). Dynamic Longitudinal Evaluation of the Utility of the Berg Balance Scale in Individuals with Motor Incomplete Spinal Cord Injury. Arch. Phys. Med. Rehabil..

[32] Kamper S.J., Maher C.G., Mackay G. (2009). Global Rating of Change Scales: A Review of Strengths and Weaknesses and Considerations for Design. J. Man. Manip. Ther..

[33] Franchignoni F., Ferriero G., Giordano A., Monticone M., Grioni G., Burger H. (2020). The Minimal Clinically-important Difference of the Prosthesis Evaluation Questionnaire—Mobility Scale in Subjects Undergoing Lower Limb Prosthetic Rehabilitation Training. Eur. J. Phys. Rehabil. Med..

[34] Schober P., Boer C., Schwarte L.A. (2018). Correlation Coefficients: Appropriate Use and Interpretation. Anesth. Analg..

[35] Mokkink L.B., Terwee C.B., Patrick D.L., Alonso J., Stratford P.W., Knol D.L., Bouter L.M., de Vet H.C. (2010). The COSMIN Checklist for Assessing the Methodological Quality of Studies on Measurement Properties of Health Status Measurement Instruments: An International Delphi Study. Qual. Life Res..

[36] Terluin B., Eekhout I., Terwee C.B. (2017). The Anchor-based Minimal important change, based on receiver operating Characteristic Analysis or Predictive Modeling, May Need to be Adjusted for the Proportion of Improved Patients. J. Clin. Epidemiol..

[37] Terwee C.B., Peipert J.D., Chapman R., Lai J.S., Terluin B., Cella D., Griffiths P., Mokkink L.B. (2021). Minimal Important Change (MIC): A Conceptual Clarification and Systematic Review of MIC Estimates of PROMIS Measures. Qual. Life Res..

[38] Terwee C.B., Bot S.D., de Boer M.R., van der Windt D.A., Knol D.L., Dekker J., Bouter L.M., de Vet H.C. (2007). Quality Criteria were Proposed for Measurement Properties of Health Status Questionnaires. J. Clin. Epidemiol..

[39] de Vet H.C., Ostelo R.W., Terwee C.B., van der Roer N., Knol D.L., Beckerman H., Boers M., Bouter L.M. (2007). Minimally Important Change Determined by a Visual Method Integrating an Anchor-based and a Distribution-based Approach. Qual. Life Res..

[40] Amsterdam Public Health [Internet] Amsterdam: Consensus-Based Standards for the Selection of Health Measurement Instruments (COSMIN). http://www.cosmin.nl.

[41] Crosby R.D., Kolotkin R.L., Williams G.R. (2003). Defining Clinically Meaningful Change in Health-related Quality of Life. J. Clin. Epidemiol..

[42] Mizuochi K. (2012). Rehabilitation Medicine in the Acute Care Setting in Japan. Japan Med. Assoc. J..

